# Foliar application of microbial consortium formulations suppresses Phomopsis leaf blight, promotes vegetative growth, and modulates biochemical defence in strawberry (*Fragaria × ananassa* Duch. cv. Camarosa) under protected cultivation

**DOI:** 10.3389/fpls.2026.1893771

**Published:** 2026-09-29

**Authors:** M K Jayavigneshwaran, Madhusmita Dishri

**Affiliations:** VIT School of Agricultural Innovations and Advanced Learning (VAIAL), Vellore Institute of Technology, Vellore, India

**Keywords:** biocontrol, carotenoid, chlorophyll, *Fragaria* × ananassa, induced systemic resistance, microbial consortium, Phomopsis leaf blight, *Phomopsis obscurans*

## Abstract

**Background:**

Strawberry (*Fragaria × ananassa* Duch. cv. Camarosa) cultivation under protected shade-net structures in South India is increasingly constrained by Phomopsis leaf blight and concurrent foliar insect infestations. Microbial formulations and consortia may provide sustainable alternatives to intensive synthetic pesticide use.

**Objectives:**

This study evaluated four commercial microbial formulations, applied individually and as a consortium at 5 and 10 mL L^−1^, for their effects on Phomopsis leaf blight severity, foliar insect incidence, vegetative growth, and biochemical traits of strawberry under shade-net conditions.

**Methods:**

Eleven treatments were arranged in a completely randomised design with three replications (132 plants). Treatments were applied after natural development of characteristic symptoms consistent with Phomopsis leaf blight. Disease and insect scores were recorded before spraying and 14 days after treatment. Vegetative and biochemical parameters were assessed two weeks after treatment. Disease severity was visually assessed using the rating approach. Data were analysed by least squares ANOVA followed by Tukey HSD at α = 0.05.

**Key results:**

The 10 mL L^−1^ complete consortium (T10) reduced disease severity to 0.00 compared with 2.56 in the untreated control and produced the highest plant spread (26.88 cm), plant height (16.33 cm), leaf area (97.37 cm²), and number of leaves (25.33). The microbial disease-control formulation at 10 mL L^−1^ (T9) produced the highest tannin, chlorophyll a, and carotenoid concentrations. The 10 mL L^−1^ treatments generally produced stronger responses than their corresponding 5 mL L^−1^ treatments.

**Conclusion:**

The complete consortium at 10 mL L^−1^ showed favourable performance in reducing symptom-based Phomopsis leaf-blight severity and improving vegetative growth. As pathogen isolation and molecular confirmation were not performed, the disease diagnosis should be regarded as symptom-based rather than definitive pathogen confirmation. Further validation is required before wider recommendation.

## 1 Introduction

Strawberry *(Fragaria × ananassa* Duch.) is one of the most economically important small fruit crops worldwide, grown on over 3,80,000 ha with an annual production of more than 9.2 million metric tonnes (Sangiorgio et al., 2023). Strawberry is not only a commercially important crop but is also recognized for its outstanding nutraceutical value, with a high content of vitamin C, anthocyanins, ellagitannins, and other bioactive phenolics that provide consumers with potent antioxidant, anti-inflammatory, and cardioprotective benefits (Martínez-Ferri et al., 2023; Raffaelli et al., 2025). The crop is commercially grown in temperate areas of Himachal Pradesh, Uttarakhand and Jammu and Kashmir, and protected cultivation technologies such as polyhouses, net-houses and shade-net structures have helped the crop to be extended to semi-arid regions like Tamil Nadu where it is also used to supplement fruit supply and diversify smallholder income (Fitriyani et al., 2025).

Although strawberry is an important horticultural and nutritional crop, protected strawberry production in South India is challenged by various biotic constraints, and Phomopsis leaf blight is an important foliar disease of strawberry. The disease has historically been associated with *Phomopsis obscurans*; however, molecular and morphological reassessment placed the strawberry leaf-blight fungus in the genus Paraphomopsis as *Paraphomopsis obscurans* (Udayanga et al., 2021). Characteristic symptoms include circular to angular, zonate lesions with dark brown to necrotic centres surrounded by reddish-purple margins on older leaves, and as the epidemic progresses, individual lesions merge to form large necrotic areas that can cover 30–60% of the leaf lamina area, significantly reducing the photosynthetically active surface and hastening canopy senescence (Abd-El-Kareem et al., 2020; Nita et al., 2003). The epidemiology of the disease is very favorable for rapid disease escalation in closed protected structures where canopy wetness occurs for long periods of time, due to conidial dispersal by rain splash and irrigation water (Abd-El-Kareem et al., 2019). At the same time, enclosed microenvironments promote the growth of sucking insect pests, such as two-spotted spider mite (*Tetranychus urticae* Koch), western flower thrips (*Frankliniella occidentalis* Pergande), and green peach aphid (*Myzus persicae* Sulzer), which can synergistically increase plant stress in combination with foliar pathogens (CABI, 2021; Yuan et al., 2023).

Microbial bioformulations, such as plant growth promoting rhizobacteria (PGPR), mycoparasites, entomopathogenic fungi and antagonistic Bacilli have become a significant force in integrated pest and disease management (IPDM) in horticultural crops. These agents provide protection to plants through various mechanisms that overlap, and all of which reduce the chance of resistance developing: (i) direct antagonism by producing cell-wall-degrading enzymes, lipopeptides (iturin A, surfactin, fengycin) and volatile organic compounds (VOCs) that inhibit fungal germination and mycelial growth; (ii) competition for infection sites and essential nutrients on leaf surfaces; (iii) elicitation of induced systemic resistance (ISR) by priming the jasmonate (JA)- and ethylene (ET)-mediated defence signalling cascades; and (iv) biostimulant activity by synthesising phytohormones such as indole-3-acetic acid (IAA), gibberellins, cytokinins, and abscisic acid modulators that enhance root architecture, nutrient uptake and photosynthetic capacity (Bejarano and Puopolo, 2020; Khan et al., 2023).

A major and under-researched area of bioagent use is the ability to achieve better efficacy from a combination of microbial formulations than from single use, and whether higher application rates give better treatment performance. Synergy cannot be assumed from a superior performance of a consortium compared to an individual formulation, however, as synergy requires an experimental design that allows comparison of the combination with appropriate combinations of the components and formal interaction analysis. The present study was not intended to provide a formal assessment of the synergistic, additive, or antagonistic interactions among the individual components of the consortium, but rather to compare the four individual microbial formulations to the four-way consortium at two application rates. Differences between individual and consortium treatments are therefore considered as differences in the performance of the treatments and not as evidence of synergism. Although the performance of each individual bioagent has been reported in strawberry crops in temperate systems (Drobek et al., 2021; Nunes et al., 2024), there is limited research on multi-component consortium applications at varying dose levels under protected cultivation conditions in tropical and semi-arid agroclimatic zones, where temperature, humidity, and radiation environments deviate significantly from temperate baselines (Dabravolski and Isayenkov, 2023; Minchev et al., 2021). Although strawberry cultivation under shade net has been gaining popularity in South India (Minchev et al., 2021; Sylla et al., 2013), to the best of our knowledge, no published studies have evaluated the use of foliar application of commercial multi component microbial consortia specifically for the management of Phomopsis leaf blight in strawberry under shade net protected cultivation in semi arid South Indian conditions. The majority of strawberry studies with PGPR are instead devoted to plant growth promotion, improvement of fruit quality, control of Botrytis cinerea or alleviation of abiotic stresses, typically in temperate or Mediterranean open field and greenhouse crops, and only a few have been conducted on Phomopsis and none under protected subtropical conditions (Hosseini et al., 2022; Mateos-Naranjo et al., 2025; Minchev et al., 2021; Raturi et al., 2023).

The objectives of the present investigation were to: (i) determine if there was any disease onset equivalence among the treatments before intervention; (ii) quantify the differential efficacy of microbial formulations (individual and consortium) applied at 5 and 10 mL L^-1^ in suppressing Phomopsis leaf blight severity and foliar insect incidence; (iii) determine the treatment-attributable effects on key vegetative growth indices: plant spread, plant height, leaf area, crown diameter and number of leaves that collectively govern canopy productivity in strawberry; and (iv) characterise the biochemical response profile of treated plants with respect to antioxidant compounds, phenylpropanoid-pathway metabolites, photosynthetic pigments, osmolyte accumulation and leaf water status. The results of this study are expected to give empirical evidence for rational design of microbial consortium spray programme to be integrated into protected strawberry IPM schedule in south India and similar agroclimatic region.

## 2 Materials and methods

### 2.1 Experimental site, crop, and growing conditions

The experiment was carried out at the VIT University research horticultural unit, Sevur, Katpadi, Vellore district, Tamil Nadu, India (12°55′N, 79°09′E; 215m above mean sea level) under 50% shade-net during the growing season of 2025 –2026. During the experimental period, the mean daily temperature, relative humidity and daily photosynthetically active radiation (PAR) under the shade-net were 18–31 °C, 58–82% and 280–540 μmol m^−2^ s^−1^, respectively. Strawberry (*Fragaria × ananassa* Duch.) cv. Camarosa was chosen as the test genotype because it is of proven commercial value and is known to be susceptible to Phomopsis leaf blight under protected conditions in humid environments. Rooted runner plants of uniform age (30 day old transplants) and vigour were established in polybags (15 L capacity) containing coco-peat: vermicompost: Rice husk (3:1:1, v/v/v). No prophylactic fungicide treatments were made during the experiment.

### 2.2 Experimental design and treatments

The experiment was set up as a completely randomised design (CRD) with 11 treatments and three replications, each with four plants (12 plants/treatment; 132 plants total). Treatments included the use of four commercial microbial formulations: combat bacteria (T_1_, T_6_), growth promoter (T_2_, T_7_), biopesticide (T3, T8), and microbial disease control (T4, T9) and their four-way consortium (T_5_, T_10_) at two concentration levels (5 and 10 mL L^-1^ of water, respectively). T_11_ was an untreated control that was water sprayed only. Details of treatments and formulation characteristics are summarised in **Table 1**. This treatment structure was used to compare individual formulations with the four-way consortium and to evaluate responses at two application rates and was not a complete factorial treatment structure to include the pairwise or three-way application combinations. Thus, the present experiment is considered a comparative treatment experiment instead of a factorial or formal interaction experiment. Thus, the terms “factorial design” and “synergistic interaction” are not used to describe the treatment structure and no formal statistical test of additive, synergistic or antagonistic interactions was conducted. Any improvement in the consortium as compared to individual formulations is considered descriptively as difference in the treatment performance. It should also be noted that in the consortium treatments each of the four components was applied at one-quarter of the corresponding single-agent dose (1.25 ml L^−1^ for each component in T_5_ and 2.5 ml L^−1^ for each component in T_10_) so that the total volume of commercial product applied in the consortium treatments equaled the corresponding single-agent dose (5 and 10 ml L^−1^, respectively). Therefore, T5 and T10 were matched to the total commercial products input for the single agent treatments, but not at the individual component level. All components were used at ¼ of the concentration used in the single agent treatments. Thus, variations in consortium versus individual treatments may be due to the fact that there are multiple microbial formulations or differences in the dose of each individual microbial component, and the performance of the consortium is not a direct measure of the equivalence or interaction of its components.

**Table 1:** Description of microbial formulation treatments applied as foliar sprays to strawberry cv. Camarosa.

Treatment	Description
T_1_	Combat bacteria, 5 ml L^−1^
T_2_	Growth-promoting bacteria, 5 ml L^−1^
T_3_	Biopesticide, 5 ml L^−1^
T_4_	Microbial disease control, 5 ml L^−1^
T_5_	Combat bacteria 1.25 ml L^−1^ + Growth-promoting bacteria 1.25 ml L^−1^ + Biopesticide 1.25 ml L^−1^ + Microbial disease control 1.25 ml L^−1^ (total 5 ml L^−1^)
T_6_	Combat bacteria, 10 ml L^−1^
T_7_	Growth-promoting bacteria, 10 ml L^−1^
T_8_	Biopesticide, 10 ml L^−1^
T_9_	Microbial disease control, 10 ml L^−1^
T_10_	Combat bacteria 2.5 ml L^−1^ + Growth-promoting bacteria 2.5 ml L^−1^ + Biopesticide 2.5 ml L^−1^ + Microbial disease control 2.5 ml L^−1^ (total 10 ml L^−1^)
T_11_	Control (water spray)

For each replication, polybag treatments were randomly allocated to positions in the shade-net, using a computer-generated random-number sequence (RAND function, Microsoft Excel) and the randomisation was done separately for each replication. The positions of polybags were fixed during the experiment. All 132 polybags were set on a single levelled bench under a single 50% shade-net canopy and fed via a common micro-sprinkler irrigation system. As there was no intentional environmental gradient, a completely randomised design (CRD) was employed (Tadesse et al., 2018). However, net-house conditions cannot entirely rule out incidental micro-environmental variations due to factors like the distance from the periphery of the shade-net, or the individual irrigation outlets. If there is a known spatial or environmental gradient, a RCBD (with blocks set up along the gradient) would be better than a CRD (Tadesse et al., 2018).

### 2.3 Spray application

The first indication of natural disease was the presence of typical symptoms of Phomopsis leaf blight on susceptible leaves 28–35 days after transplanting. Foliar spray applications of all formulations were made in the early morning (06:00–08:00 h). This is due to the rationale of the practical application, not an experimental mechanism for the specific formulations used in this study, and is common for foliar applied microbial biopesticides and biostimulants, which are known to be sensitive to solar UV radiation and desiccation, and where the naturally higher moisture on leaf surfaces during the early morning is thought to be beneficial for microbial attachment and establishment on the phyllosphere (Lindow and Brandl, 2003; Tadesse Mawcha et al., 2025). Each formulation was freshly diluted in tap water (pH 6.8-7.0) to the desired concentration just prior to application and applied with a calibrated backpack sprayer (0.5 L nozzle discharge per plant; uniform canopy coverage to run-off). An equal amount of water was applied to control plants. To avoid cross contamination, all spray equipment was surface sterilised with 70% ethanol between treatments.

### 2.4 Disease and insect assessment

Two disease severity and insect incidence assessments were made: one immediately prior to spray application (pre-spray baseline) and one 14 days after spray application (post-spray). Disease severity was visually assessed on 5 randomly selected fully expanded mid-canopy leaflets per plant using an ordinal rating scale described by Nita et al. (2003) for Phomopsis leaf blight with each grade representing a specified percentage of leaf lamina area with diagnostic lesion symptoms (circular to zonate, dark brown necrotic lesions with reddish-purple margins): 0 = no visible lesions; 1 = 1-10% of leaf area affected; 2 = 11-25%; 3 = 26-50%; 4 = 51-75%; and 5 = >75% of leaf area affected. A mean disease rating per plant was determined and the mean of four plants per replication was used for analysis. In the field, the general morphology of the insects was examined using a 10× hand lens, and the predominant arthropod pests of the experimental plants were the two-spotted spider mite (*Tetranychus urticae* Koch), western flower thrips (*Frankliniella occidentalis* Pergande), and green peach aphid (*Myzus persicae* Sulzer). A 14-day ordinal scale was used to assess each leaflet for these three taxa (mite, thrips and aphid) because these taxa occurred together on the same leaflets and caused overlapping feeding symptoms (stippling/bronzing caused by mites, silvering/scarring caused by thrips, and honeydew-associated sooty mould caused by aphids); this composite scale was similar to the pest guild level approach used in strawberry arthropod pest management, and reflects overall canopy level pest pressure rather than the incidence, abundance and severity of the feeding damage caused by each taxon individually. The incidence of foliar insects was assessed on the same plants by the percentage of leaves with insect feeding damage and the apparent severity of the visible insect damage, according to the scouting procedures outlined by Lahiri et al. (2022). Insect and disease scores were taken as replication means.

### 2.5 Vegetative growth parameters

At 14 days after spray, vegetative assessments were made on all four experimental plants per replication. The horizontal spread of the plant was measured as the maximum diameter of the canopy (cm). Plant height was measured from the bottom of the crown to the top of the longest fully expanded leaf (cm). Leaf area was calculated by gravimetric disc method using a standard lamina punch with known area (1 cm²) and total fresh weight of leaf discs (FWLD) to whole leaf fresh weight. Crown diameter was measured at the maximum cross section of the crown with a digital Vernier calliper with a precision of ±0.01 mm. The number of fully expanded leaves per plant and number of runners per plant were determined by direct count.

### 2.6 Biochemical analyses

Immediately after the vegetative observations, two representative plants were sampled from each replication, leaves were pooled within replication, snap frozen in liquid nitrogen and stored at −80 °C until analysis. Pooling of leaf tissue from the two sampled plants was used to provide enough fresh leaf mass for the full suite of biochemical assays (photosynthetic pigments, total phenol, flavonoid, tannin, DPPH, FRAP, proline, protein and relative water content) to be performed in triplicate from a single homogeneous leaf sample, rather than as a replacement for biological replication of individual plants. Leaf material from the two plants was pooled prior to extraction within each replication, so the biochemical values reported are the average biochemical status of the plants sampled within each replication and biological (plant-to-plant) variability was not estimated from the present data; rather the triplicate analytical measurements performed on each pooled sample reflect analytical (measurement) precision. All biochemical assays were carried out in triplicates on fresh weight (FW) basis.

#### 2.6.1 Photosynthetic pigments (chlorophyll a, chlorophyll b, carotenoids)

The acetone extraction method was used to determine the levels of chlorophyll a, chlorophyll b and total carotenoid contents. Fresh leaf tissue (0.5 g) was ground in a pre-chilled mortar with 10 mL of 80% (v/v) acetone and a pinch of MgCO_3_ (to prevent pheophytinisation). The homogenate was filtered through Whatman No. 1 filter paper and the volume made up to 25 mL with 80% acetone. The absorbance of the clarified extract was measured spectrophotometrically at 663, 645 and 470 nm with a UV-Vis spectrophotometer. The concentration of chlorophyll a, chlorophyll b and carotenoid were calculated according to the equations of Lichtenthaler (1987) as follows:


$$Chl\;a\;\left(mg\;g^{-1}\;FW\right)=\left(12.21\;\times \;A663\;-\;2.81\;\times \;A645\right)\;\times \;V/\left(1000\;\times \;W\right)$$

$$Chl\;b\;\left(mg\;g^{-1}\;FW\right)=\left(20.13\;\times \;A645\;-\;5.03\;\times \;A663\right)\;\times \;V/\left(1000\;\times \;W\right)$$

$$Carotenoids\;\left(mg\;g^{-1}\;FW\right)=\left(1000\;\times \;A470\;-\;3.27\;\times \;Chl\;a\;-\;104\;\times \;Chl\;b\right)/229\;\times \;V/\left(1000\;\times \;W\right)$$

where V = total extract volume (mL) and W = fresh weight of tissue (g).

Total phenol: The total phenolic content was measured by the Folin-Ciocalteu colorimetric method (Swain and Hillis, 1959) and reported as mg gallic acid equivalents (GAE) g^-1^ FW. Total flavonoid: The amount of flavonoids was determined by the aluminium chloride (AlCl_3_) colorimetric method (Zhishen et al., 1999) using rutin as standard and the absorbance was measured at 510 nm. Tannin: The condensed tannin (proanthocyanidin) content was determined by the vanillin-HCl method with catechin as standard (Julkunen-Tiitto, 1985) and expressed as mg catechin equivalents (CE) g^−1^ FW. DPPH radical-scavenging activity: The free radical scavenging capacity was determined by 2,2-diphenyl-1-picrylhydrazyl (DPPH) and reported as % inhibition of DPPH radical compared to a methanol blank. Ferric reducing antioxidant power (FRAP): The FRAP was measured using the method of Benzie and Strain (1996) and the results were presented as mM ascorbic acid equivalents (AAE) g^-1^ FW. Free proline: The proline was determined by ninhydrin colorimetric method of (Bates et al., 1973) in the extracts obtained by sulfosalicylic acid and the absorbance obtained at 520 nm and expressed as μmol g^-1^FW. Protein: Total soluble protein was measured by the method of (Lowry et al., 1951) with bovine serum albumin (BSA) as standard, absorbance was measured at 660 nm and results were expressed as mg g^-1^ FW. Relative water content (RWC): The RWC of leaf discs was calculated as per Ievinsh (2023) by using the formula: $RWC=\frac{FW-DW}{TW-DW}\times 100$

Where FW = fresh weight of the leaf disc (g) recorded immediately after sampling, TW = turgid weight (g) recorded after rehydrating the leaf disc to full turgidity, and DW = dry weight (g) recorded after oven-drying the leaf disc to a constant weight.

### 2.7 Statistical analysis

Least squares fit model was used to analyse all response variables in JMP^®^ statistical software (Version 17, SAS Institute Inc., Cary, NC, USA). Analysis of variance (ANOVA) was used to determine the treatment and replication effects. When F-ratios were significant (p ≤ 0.05), the treatment means were separated by the Tukey-Kramer Honest Significant Difference (HSD) test at α = 0.05. Logworth (−log_10_(p)) values are provided for effect summaries in addition to p-values. R² and adjusted R² were used to measure the goodness of fit of the models. The severity of disease and insects were evaluated by ordinal rating scale, and the mean of the observations made on the five leaflets per plant and four plants per replication was taken as the observation for the experimental unit. Therefore, each value at a replication level was the result of several observations, instead of a single leaflet or plant being considered as the experimental unit. When the mean of the experimental unit derived from the ordinal or percentage class disease assessment is an appropriate summary of the disease and model assumptions are satisfied, then mean disease data has been analysed using parametric methods (Beruski et al., 2016; Nutter and Schultz, 1995). The diagnostic plots and tests produced by the JMP^®^ Fit Model procedure were used to check for residual normality and homogeneity of variance for responses analysed by parametric procedures. The Tukey-Kramer honestly significant difference (HSD) test at α = 0.05 was used to compare treatment means, when appropriate.

## 3 Results

### 3.1 Pre-spray baseline assessment of disease and insect scores

Disease and insect ratings were made on all experimental plants prior to spray treatments. Results indicated that there were no significant differences between the insect score or disease score of the various treatments at the time of baseline recording. The grand mean of insect score before spray was 2.78 and the overall model was not significant. There were also no significant differences (p > 0.05) in disease score prior to spray. These results verify that all treatment groups were subjected to the same biotic stress conditions at the beginning of the experiment and that any differences noted after the spray application were due to the treatments.

### 3.2 Effect of treatments on post-spray disease and insect scores

Disease score was significantly different among treatments after 14 days of spray application (p ≤ 0.01). Insect score showed a borderline overall model significance, with individual treatment contrasts for T_2_ (p = 0.0242) and T_3_ (p = 0.0484) reaching significance. **Table 2** shows the data. The post-spray disease score was completely suppressed with T_9_ (microbial disease control at 10 mL L^-1^) and T10 (full consortium at 10 mL L^-1^), whereas the untreated control (T_11_) had a disease score of 2.56. The next best reduction was seen in the post-spray disease scores of T_5_ (consortium) and T_4_ (microbial disease control) with scores of 0.56 and 0.67 respectively among the 5 mL L^-1^ treatments. Moderate reduction was seen with single agent low dose treatments. The post spray mean insect score for all treatments was 2.23 compared to a pre spray mean of 2.78, showing a general suppressive trend with the application of treatment.

**Table 2:** Effect of microbial formulation treatments on insect and disease scores before and after foliar spray application in strawberry cv. Camarosa.

Treatment	Insect score		Disease score	
	Before spray	After spray	Before spray	After spray
T_1_	2.33	1.33	2.56	0.89
T_2_	2.86	1.86	3.22	1.56
T_3_	2.75	1.75	3.11	1.11
T_4_	2.58	1.58	2.89	0.67
T_5_	2.78	1.78	2.78	0.56
T_6_	2.17	1.17	2.44	0.22
T_7_	2.22	1.56	2.33	0.33
T_8_	2.75	1.75	2.89	0.67
T_9_	2.22	1.56	2.22	0.00
T_10_	2.22	1.22	2.22	0.00
T_11_ (Control)	2.78	2.78	3.56	2.56
SE ±	0.248	0.248	0.248	0.248
p-value	NS	*	NS	**

Values are means of three replications. For after-spray values, means within a column followed by the same letter are not significantly different (Tukey HSD, α = 0.05). NS, non-significant; *p ≤ 0.05; **p ≤ 0.01.

### 3.3 Effect of treatments on vegetative growth parameters

Plant spread, plant height, leaf area and number of leaves per plant were highly significantly (p ≤ 0.01) affected by treatment. There were no significant differences between treatments for runners per plant and crown diameter (p > 0.05). The data are shown in **Table 3**.

**Table 3:** Effect of microbial formulation treatments on vegetative growth parameters of strawberry cv. Camarosa assessed 14 days after foliar spray application.

Treatment	Runners plant^−1^	Plant spread (cm)	Plant height (cm)	Leaf area (cm²)	Crown diameter (cm)	No. of leaves
T_1_	2.33	24.67 b	11.33 d	46.40 e	1.17	19.00 c
T_2_	2.33	23.52 bc	11.67 d	54.87 d	1.33	19.67 c
T_3_	2.67	24.97 b	13.00 c	67.41 c	1.50	21.67 b
T_4_	2.67	24.06 b	12.00 d	77.44 b	1.33	20.00 b
T_5_	2.67	24.37 b	13.33 c	84.41 b	1.50	20.67 b
T_6_	2.33	22.60 c	11.67 d	43.41 e	1.17	18.67 c
T_7_	2.33	22.61 c	12.00 d	56.19 d	1.33	19.33 c
T_8_	2.67	23.61 bc	12.67 d	69.40 c	1.33	20.67 b
T_9_	2.67	25.06 b	13.67 c	78.16 b	1.50	22.00 b
T_10_	3.00	26.88 a	16.33 a	97.37 a	1.67	25.33 a
T_11_	2.00	19.28 d	10.33 e	30.28 f	1.00	16.33 d
SE ±	0.19	0.68	0.44	3.12	0.09	0.70
p-value	NS	**	**	**	NS	**

Values are means of three replications. Means within a column followed by the same letter are not significantly different (Tukey HSD, α = 0.05). NS, non-significant; **p ≤ 0.01.

Plant spread: T_10_ (26.88 cm) had the greatest plant spread and was significantly better than all other treatments (Tukey group a). T9 (25.06 cm) and T3 (24.97 cm) were next best (group b). The plant spread of the untreated control T_11_ was the lowest (group d) at 19.28 cm. The model R² for plant spread was 0.791, which was high and showed strong influence of treatment.

Plant height: T10 had the tallest plants (16.33 cm) and was in a distinct superior Tukey group (a). T_9_ (13.67 cm) and T5 (13.33 cm) were in the next best groups. The shortest plants (10.33 cm, group e) were found in the control T_11_. The 10 mL L^−1^ treatments generally gave a higher growth response than the 5 mL L^−1^ treatments within the treatments evaluated.

Leaf area: Leaf area was the most sensitive to treatment with eleven Tukey groups (a to k) separating the eleven treatments. The leaf area of T_10_ was the highest (97.37 cm²) and the lowest was found in the control (30.28 cm²) (group f). T_5_ (84.41 cm²) and T_9_ (78.16 cm²) were in group b. The individual treatments and the consortium were higher ranked groups.

Number of leaves: T10 had the highest number of leaves per plant (25.33) and was in a separate superior group (a). T_9_ (22.00) and T_3_ (21.67) followed in group b. T_11_ had the fewest leaves (16.33, group d).

Crown diameter and runners: No significant differences were observed among treatments for crown diameter and number of runners per plant, but there was a numerical increase in both parameters in T10 compared to the control.

### 3.4 Effect of treatments on biochemical parameters

#### 3.4.1 Antioxidant parameters, tannin, proline, and protein

Among the seven parameters in this group, tannin content was the only one that showed a significant treatment effect (p ≤ 0.05). No significant differences (p > 0.05) were observed among the treatments for total phenol, DPPH, FRAP, proline and protein. Total phenol content was significantly affected by replication (p = 0.0035), which suggests some spatial variation within the shade-net structure. The data is given in **Table 4**. T_9_ had the highest tannin content (4.82 mg CE g^-1^ FW, group a) which was significantly higher than the control T_11_ (3.43 mg CE g^-1^ FW, group c). T_10_ (4.28 mg CE g^−1^ FW) and T6 (4.02 mg CE g^−1^ FW) were in intermediate group ab. The majority of the 5 mL L^-1^ treatments were in group bc. T_9_ also had the highest total phenol (1.20 mg GAE g^-1^ FW) and DPPH (52.46% inhibition) than other treatments, but these were not significantly different.

**Table 4:** Effect of microbial formulation treatments on antioxidant and phenolic parameters in strawberry cv. Camarosa leaves.

Treatment	Total phenol (mg GAE g^−1^ FW)	DPPH (% inhibition)	FRAP (mM AAE g^−1^ FW)	Tannin (mg CE g^−1^ FW)	Proline (μmol g^−1^ FW)	Protein (mg g^−1^ FW)
T_1_	0.82	47.62	1.82	3.57 bc	0.31	0.32
T_2_	0.76	47.24	1.81	3.78 bc	0.28	0.33
T_3_	0.86	47.37	1.82	3.57 bc	0.30	0.34
T_4_	0.78	47.22	1.71	3.44 bc	0.32	0.30
T_5_	0.85	46.99	1.80	3.67 bc	0.29	0.34
T_6_	0.90	48.83	1.81	4.02 ab	0.34	0.35
T_7_	0.88	47.49	1.82	3.92 bc	0.33	0.35
T_8_	0.79	47.14	1.81	3.57 bc	0.30	0.33
T_9_	1.20	52.46	1.88	4.82 a	0.41	0.39
T_10_	0.91	48.47	1.84	4.28 ab	0.33	0.35
T_11_	0.89	47.62	1.80	3.43 c	0.29	0.32
SE ±	0.09	1.23	0.05	0.25	0.03	0.02
p-value	NS	NS	NS	*	NS	NS

GAE, gallic acid equivalents; CE, catechin equivalents; AAE, ascorbic acid equivalents; FW, fresh weight; NS, non-significant; *p ≤ 0.05 (Tukey HSD, α = 0.05).

#### 3.4.2 Photosynthetic pigments and relative water content

The content of chlorophyll a and carotenoids was significantly different between treatments (p ≤ 0.05). The treatments did not significantly affect chlorophyll b and relative water content (RWC) (p > 0.05). The extraction of all pigments was done in 80% acetone and the absorbance was measured at 663, 645 and 470 nm using (Lichtenthaler, 1987) equations. **Table 5** shows data. T9 had the highest chlorophyll a content (2.39 mg g^−1^ FW, group a), significantly higher than the control T11 (1.84 mg g^−1^ FW, group b). All other treatments were in the intermediate group ab. T_9_ also exhibited the highest carotenoid content of 1.01 mg g-1 FW (group a) which was significantly different from T11 (0.82 mg g-1 FW, group b). The chlorophyll b content varied from 1.93 (T_7_) to 2.22 mg g-1 FW (T_9_) and did not differ significantly among the treatments. RWC was consistent in all the treatments ranging from 96.92% to 97.86% and was not significantly affected by the treatments.

**Table 5:** Effect of microbial formulation treatments on photosynthetic pigments (80% acetone extraction) and relative water content in leaves of strawberry cv. Camarosa.

Treatment	Chl a (mg g^−1^ FW)	Chl b (mg g^−1^ FW)	Carotenoid (mg g^−1^ FW)	RWC (%)
T_1_	2.14 ab	2.07 ab	0.88 ab	97.86
T_2_	2.14 ab	2.00 ab	0.97 ab	96.92
T_3_	2.11 ab	2.09 ab	0.88 ab	97.05
T_4_	2.17 ab	2.10 ab	0.96 ab	97.61
T_5_	1.94 ab	2.06 ab	0.94 ab	97.21
T_6_	2.15 ab	2.06 ab	0.92 ab	97.46
T_7_	1.93 ab	2.11 ab	0.95 ab	97.38
T_8_	1.88 ab	2.04 ab	0.92 ab	97.07
T_9_	2.39 a	2.22 ab	1.01 a	97.74
T_10_	2.04 ab	2.06 ab	0.98 ab	97.51
T_11_	1.84 b	1.96 ab	0.82 b	97.00
SE ±	0.13	0.09	0.05	0.44
p-value	*	NS	*	NS

FW, fresh weight; NS, non-significant; *p ≤ 0.05 (Tukey HSD, α = 0.05).

## 4 Discussion

### 4.1 Baseline homogeneity validates experimental design

The fact that there were no significant differences between the eleven treatment groups in pre-spray insect and disease scores is an important confirmation that the experimental units were homogeneous at the beginning of the trial. This similarity of initial conditions is a fundamental condition for any comparative biocontrol experiment to ensure that any differences after the treatments are applied are attributable to the treatments and not to the initial differences in pest or disease pressure (Cremonez et al., 2023). In a similar manner, (Zeinalipour and Saadati, 2024) conducted a factorial experiment on strawberry and validated the initial physiological parameters of the treatments, which were found to be equal before any foliar or soil application was made, thereby enhancing the reliability of comparing the treatments.

### 4.2 Mechanisms of disease suppression and insect score reduction

The disease score of 2.56 for the untreated control is significantly higher than the scores of 0.00 for T9 and T10 (both total product inputs of 10 mL L^−1^), demonstrating the strong disease-suppressive effect of the microbial disease-control formulation and the complete consortium at 10 mL L^−1^ total product input. The biocontrol microorganisms in these formulations act in several ways to control foliar fungal pathogens. These involve direct production of antifungal compounds (e.g., lipopeptides like iturin A, fengycin and surfactin in Bacillus-type agents), competition for nutrients and colonisation sites on the leaf surface, and indirect activation of plant immune responses called induced systemic resistance (ISR) (Balleux et al., 2025). ISR is mediated via the jasmonate and ethylene signalling pathway, which increases the expression of pathogenesis-related (PR) proteins such as chitinase and β-1,3-glucanase, which enhances cell wall integrity and slows the progression of fungi (Han and Schneiter, 2024). The higher disease suppression seen with the 10 mL L^−1^ treatments compared to the corresponding 5 mL L^−1^ treatments indicates better treatment activity at the higher rate tested. The production of antifungal metabolites and ISR elicitors might not be enough to induce a measurable protective response within the 14 days observation period of this study, below a minimum cell density threshold (Legein et al., 2020; Zhou et al., 2018). This threshold colonisation concept is well documented in biocontrol literature and is a useful practical guideline to use higher application rates to achieve better and more consistent disease suppression (Riseh et al., 2025). The borderline model significance of post-spray insect score (p = 0.086) and the individual significance for T_2_ and T_3_ at 5 mL L^-1^ suggest that the microbial formulations had a moderate and variable effect on insect pressure during the short assessment period. The slightly higher score for insects in T_2_ and T_3_ than the mean could be due to the temporary increase in leaf attractiveness as a result of the succulence caused by the auxin produced by the PGPR in young, rapidly growing leaves as reported by (Cardarelli et al., 2024; Paliwoda et al., 2022) in strawberry plants grown at sub-optimal concentrations of auxin-producing PGPR. The lower insect scores at higher doses, especially T_9_ and T_10_, may be related to the higher amount of tannins found in these plants, which make the foliage less palatable to phytophagous insects (Barbehenn and Peter Constabel, 2011; War et al., 2012). Insect damage in the present study was reported as a composite guild-level index for mites, thrips and aphids so that the response of each pest taxon could not be determined separately. Future trials could evaluate the impact of microbial treatments on each member of the pest guild by making a taxon-specific assessment of pest incidence, abundance (e.g., count per leaf or trap catch), and damage severity.

### 4.3 Growth promotion through microbial phytohormone and nutrient mechanisms

The significant differences in all four key vegetative parameters (plant spread, plant height, leaf area and number of leaves) between T_10_ and the control confirms the strong biostimulant effect of consortium microbial formulations on strawberry plant growth. The growth promotion of plants by PGPR is a well-studied phenomenon, which is mediated by the production of phytohormones like indole-3-acetic acid (IAA), cytokinins and gibberellins, and also by enhancing the availability of nutrients through phosphate solubilisation and biological nitrogen fixation (Agbodjato and Babalola, 2024; Castiglione et al., 2021). The growth promoting activity of the four functionally complementary microorganisms in a consortium as in T10, together with other consortium members, can affect plant growth in several ways such as production of phytohormones, nutrient mobilization and modulation of plant defence (Ehinmitan et al., 2024; Suriani et al., 2025). In the present experiment, however, only the four individual formulations and the complete four-way consortium were used, and the four pairwise and three-way combinations were not included; thus, the relative contribution of individual components and interactions cannot be statistically separated. Thus, it is not considered statistically demonstrated synergistic interaction, but a consortium effect that is better seen in the growth response of T10.

Plant spread and height: T_10_ showed 39.4% and 58.1% increase in plant spread and plant height respectively, as compared to the control T_11_. The IAA produced by PGPR strains in the consortium stimulate the elongation of petiole by stimulating the expansion of cells in the cortex parenchyma, while gibberellins stimulate the elongation of internodes which help in the increase of plant height (Khoso et al., 2024; Raturi et al., 2023). The observed effect on T10 could be due to the overall effect of the microbial consortium on the growth of the plants but the effect of individual components of the microbial consortium or their interactions cannot be separated in the present treatment structure. Similar increases in plant height and canopy spread of strawberry plants after PGPR consortium application were observed by (W. Sun et al., 2024), which was attributed to the effect of IAA and gibberellin on cell elongation in petioles and crown tissue.

Leaf Area: The most remarkable vegetative outcome of this study is the three fold increase in leaf area from 30.28 cm² (T_11_) to 97.37 cm² (T_10_). Leaf area is the key factor to determine the whole-plant photosynthetic rate and carbon assimilation in strawberry, and its improvement can directly promote the productivity of the later growth stages (Gupta et al., 2022; Luo et al., 2024). The consortium contains bacteria that produce cytokinins which stimulate cell division in developing leaf primordia, thus helping to increase the area of the lamina, and IAA which stimulates the cell elongation component of leaf growth. (Todeschini et al., 2018) also found that strawberry plants inoculated with beneficial microorganisms had a significantly larger leaf area, especially when more than one group of microorganisms with complementary mechanisms was used. The separation between the treatments for leaf area suggests that this parameter was sensitive to the microbial formulations and rates tested in the present study. The higher leaf area recorded with T10 is due to the formulation of the tested consortium under the experimental conditions and does not necessarily indicate synergistic interaction between the components of the consortium.

Number of leaves: The number of leaves in T_10_ (25.33) and T9 (22.00) was higher than T_11_ (16.33) which is indicative of PGPR mediated promotion of axillary bud break and faster plastochron rate. The microbial consortium produces cytokinins, which helps to decrease the apical dominance and promote outgrowth of lateral buds in the strawberry crown, thus stimulating new leaf initiation rate (Andrés et al., 2021; Ferguson and Beveridge, 2009). Increased number of functional leaves leads to a higher photosynthetically active surface area of the plant, and consequently, more assimilate production for vegetative growth and subsequent fruit development (Pérez-Rojas et al., 2023).

Crown diameter and runners: The lack of significance for these two parameters is biologically logical since they were measured 14 days after a single spray application. Crown expansion is a slow developmental process and is not likely to show measurable differences between treatments within 2 weeks of foliar application, as several rounds of cell division and differentiation at the crown growing point are required. In strawberry, the production of runners is mainly influenced by photoperiod and temperature and is not very sensitive to biostimulant treatment in short experimental periods (Li et al., 2020; Yang et al., 2024).

### 4.4 Biochemical defence and photosynthetic pigment responses

#### 4.4.1 Tannin: the key biochemical response

In this study, only tannin was a statistically significant treatment effect parameter related to antioxidants. The highest tannin accumulation in T_9_ (4.82 mg CE g^-1^ FW) as compared to the control T_11_ (3.43 mg CE g^-1^ FW) clearly indicates that the microbial disease control agent at 10 mL L^-1^ was most effective in inducing the phenylpropanoid defence pathway in strawberry leaves. Condensed tannins are formed by the flavonoid branch of the phenylpropanoid pathway, and the enzyme phenylalanine ammonia-lyase (PAL) is a key enzyme in the regulation of this pathway. Biocontrol microorganisms colonising the phyllosphere induce greater metabolic flux towards the production of proanthocyanidins and condensed tannins in the leaf tissue by inducing jasmonate (JA)-responsive transcription of PAL and downstream enzymes (Singh et al., 2010; Zhang et al., 2025).

Tannins can also play a role in strengthening physical and biochemical barriers and in regulating defence-associated processes, which can lead to an increase in plant resistance to foliar pathogens (Barbehenn and Peter Constabel, 2011; Singh et al., 2010). In the present study, however, the penetration of pathogens, infection structures and tissue colonization were not directly evaluated, and the observed correlation between the severity of Phomopsis leaf-blight and tannin concentration should be considered as a possible defence-related response, but not a proof of a tannin-mediated mechanism. The high tannin content found in T9 could be responsible for its relatively low disease severity, but this needs to be tested experimentally.

Tannins can also play a role in plant defence against phytophagous insects via feeding deterrence, digestive physiology, and other biochemical interactions in the insect gut (Barbehenn and Peter Constabel, 2011; War et al., 2012). Traditionally, tannins have been suggested to affect herbivore performance by binding proteins and disrupting digestion, but they can also have oxidative and other biochemical effects, and their impacts on herbivore performance will depend on the structure of the tannin and the species of the insect (Barbehenn and Peter Constabel, 2011). The lower insect scores observed in T9 and T10 than in the untreated control are thus consistent with a possible contribution of tannin-associated plant defence, but should be treated with a degree of caution, as differences in insect scores between treatments were not statistically significant.

The dose dependent tannin response (T_9_ (10 mL L^-1^) significantly higher than T_4_ (5 mL L^-1^; 3.44 mg CE g^-1^ FW) indicates that a minimum effective concentration of the bioformulation is needed to stimulate the ISR mediated tannin biosynthesis pathway enough to see a difference. Similarly, (Mora et al., 2022; Mukadam et al., 2024; Rahman et al., 2018) reported that Bacillus-based bioformulations induced the production of phenylpropanoid compounds in strawberry in a dose-dependent manner, with the highest doses yielding the most robust and consistent biochemical defense responses.

#### 4.4.2 Total phenol, DPPH, FRAP, proline, and protein

The lack of significant treatment effect on total phenol, DPPH, FRAP, proline and protein is informative but not surprising, and can be explained by taking into account the nature of these metabolites and the experimental conditions.

Total phenol content was significantly affected by replication (p = 0.0035), but not by treatment (p = 0.137). The strong replication influence suggests that the spatial variation in light intensity within the shade-net structure where plants in different rows receive slightly different photosynthetically active radiation (PAR) was more important than the treatments in the 14-day study period. UV-B and visible light have a strong effect on phenolic biosynthesis in strawberry; even minor differences in PAR exposure in a protected structure can result in large differences in phenolic concentrations. (Liu et al., 2025; Milosavljević et al., 2022) also noted that sometimes, the environmental conditions in a controlled growing structure had a greater effect on biochemical parameters than the treatments and suggested that a longer period of treatment or multi-spray programmes would be needed to see the biochemical response to treatments clearly. The non-significance of the DPPH and FRAP antioxidant assays can be directly attributed to the non-significance of total phenol because the antioxidant activity of plant extracts is largely dependent on the phenolic pool available.

Proline and Protein contents were also not influenced by treatments as would be expected under the experimental conditions. Proline is mainly accumulated in plants tissues as an osmotic adjustment mechanism during drought, salinity or heat stress (Raza et al., 2023). In this study, no plants were intentionally stressed and all plants were grown under well-irrigated, temperature moderated conditions inside the shade-net structure. In such favourable growing conditions, the level of proline is low and does not respond significantly to biostimulant treatment as also reported by Al-Turki et al. (2023) in their review on the effects of PGPR on proline metabolism. In a similar way, total soluble protein does not change significantly in non-stressed leaves and is relatively stable under optimal conditions after short duration biostimulant treatments.

#### 4.4.3 Chlorophyll a, chlorophyll b, carotenoid, and relative water content

Chlorophyll a: The significantly higher chlorophyll a content in T_9_ (2.39 mg g^−1^ FW) compared to the control T_11_ (1.84 mg g^−1^ FW) shows that the microbial disease control formulation at 10 mL L^−1^ enhanced the leaf photosynthetic pigment status. The amount of chlorophyll a is directly related to the photosynthetic capacity of the leaf and is the main photoreactive pigment of both Photosystem I and Photosystem II in the thylakoid membrane (Petibon and Wiesenberg, 2022). This enhancement can be attributed to three mechanisms. First, T_9_ plants did not have any active Phomopsis infection in them, which in the control plants leads to ROS mediated oxidative damage to chloroplasts, accelerating chlorophyll degradation by chlorophyllase activity. T_9_ maintained the integrity of chloroplast and the existing pool of chlorophyll a by suppressing the pathogen (Wang et al., 2023). Second, the PGPR strains in the biocontrol formulation could have increased the availability of Mg^2+^ via rhizospheric acidification, which could have increased the availability of more substrate for *de novo* tetrapyrrole synthesis in the chloroplasts (Alzate Zuluaga et al., 2024). Third, the bacteria in the consortium are known to produce cytokinin, which is known to delay leaf senescence by reducing the expression of chlorophyllase genes, and thus prolong the pool of chlorophyll a in the treated plants (Wu et al., 2021).

Carotenoid: The physiological finding of the significantly higher carotenoid content in T_9_ (1.01 mg g^-1^ FW) than in T11 (0.82 mg g^-1^ FW) is important. The carotenoids in the chloroplast have two major roles: they are accessory light-harvesting pigments which absorb light in the blue-green region (450–490 nm) and transfer the energy to chlorophyll a for photosynthesis, and they protect the photosynthetic apparatus from excess excitation energy by non-photochemical quenching (NPQ) (Hashimoto et al., 2016; Sun et al., 2022). The increase in carotenoid content in T_9_ plants under the 50% shade-net conditions of this experiment may be a photo acclimation response to maximise the efficiency of light capture per unit leaf area, since the amount of incident PAR was significantly reduced. The simultaneous increase of both chlorophyll a and carotenoid in T_9_ may be indicative of the coordinated assembly of these pigments in the photosynthetic membrane complexes of the chloroplast, since both are needed to assemble the reaction centre and antenna complexes for their structural and functional integrity (Quian-Ulloa and Stange, 2021).

Chlorophyll b: There was no significant difference among the treatments and T9 had the highest value numerically (2.22 mg g^-1^ FW). The light-harvesting complex II (LHCII) is the site of chlorophyll b, and it is a relatively stable structure in the thylakoid membrane, with a relatively slow turnover of pigments compared to the reaction-centre chlorophyll a pool. The differential significance between chl a and chl b in this study is explained by the fact that short-term biostimulant treatments within a 14-day window primarily affect the dynamic, repair dependent chlorophyll a pool and not the more stable LHCII associated chlorophyll b (Lingvay et al., 2020; Mao et al., 2025).

Relative Water Content: RWC was not significantly different among the treatments and was high (96.92-97.86%). This indicates that the plants were well watered during the study period under the drip-irrigated shade-net growing conditions employed. Similarly, (Aathithyan et al., 2025) found that the differences between the treatments in RWC were only observed when the plants were subjected to deficit irrigation and not under well-watered conditions. The uniformity of RWC in the current study thus confirms that the water stress was not different between the treatments and the significant differences in chlorophyll a, carotenoid, tannin and vegetative parameters were not due to differential water stress.

### 4.5 Comparative performance of treatments

Overall, T10 exhibited the best vegetative growth parameters, while T9 exhibited the best disease suppression and selected biochemical traits. This meant that the relative effectiveness of these treatments was dependent on the response variable measured and not a treatment ranking. T10 performed best for plant spread, plant height, leaf area and number of leaves, and T9 had the best disease-suppressive response and higher levels of tannin and selected photosynthetic pigments. The results suggest that the treatments had differing effects on the different response variables tested in this experiment.

The overall higher performance of the 10 mL L^−1^ treatments, and the positive performance of the full consortium compared to some individual formulations, should be considered within the framework of the treatment structure used in this study. The improvements seen with T10 could be attributed to the combined effect of the four microbial formulations on plant growth, disease control and physiological reactions, as microbial consortia have been shown to have a multifaceted effect on plant growth in other crops (Negi et al., 2024). The contributions and interactions of consortium components, however, cannot be separated because the experiment was not conducted with the corresponding pairwise and three-way combinations. Thus, the performance of T10 observed does not necessarily constitute proof of synergistic action but rather improved efficacy of the tested consortium formulation. The same improvement in plant growth after microbial consortia application has been reported in other cropping systems (Negi et al., 2024).

The microbial consortium developed here performed well in total product input at 10 mL L^-1^ and may be beneficial for incorporation into IPM programmes for protected strawberry production, as other microbial consortia have been reported to enhance plant growth and disease management by complementary microbial functions (Negi et al., 2024). This is a recommended practice but should be treated as a preliminary recommendation until validated seasonally, geographically and under production conditions.

## 5 Limitations

This study was conducted in one season at one experimental location in a shade-net environment and the uniformity of the responses found across seasons, locations, environmental conditions, and strawberry production systems has not been determined. The study was mainly based on disease severity, vegetative growth and selected biochemical responses, while fruit yield and quality attributes such as total soluble solids, firmness, colour, and other market and consumer relevant attributes were not assessed. The microbial formulations were also not evaluated for residue or economic considerations. Disease assessment was restricted to pre-spray and 14 days after spraying. Therefore, it was not possible to adequately describe the temporal course of the disease and an area under the disease progress curve (AUDPC) could not be determined from the data collected. The insect assessment was also included as a composite index of mites, thrips and aphids, so the relative importance of individual pest taxa in the observed treatment responses could not be separated. Incidence, abundance and damage severity were not measured separately for each taxon.

One further constraint is that the causal organism of the symptoms of leaf-blight observed in the field was not independently confirmed by pathogen isolation, culture based morphological characterization or molecular sequencing. Diagnosis of disease was made based on typical symptoms and visual disease assessment by following a standard rating system for Phomopsis leaf blight. The disease diagnosis should thus be considered as a symptom diagnosis and not as a definitive diagnosis of the causal pathogen. Four individual microbial treatments and their full 4-way consortium were included in the treatment structure, but not the corresponding pairwise and three-way combinations. Therefore, the relative performance of the entire consortium is not a good indicator of additive, synergistic, or antagonistic interactions between the microorganisms. Furthermore, the total commercial product input was the same for consortium treatments as it was for the individual treatments, but each component of the consortium was used at one-fourth of the concentration of the individual treatment. Therefore, any differences between treatments may be due to the formulation composition and/or the dose of the components. Leaves from 2 representative plants in each replication were combined to yield a composite sample for biochemical analyses. Pooled samples were considered one biological replicate and analytical triplicates were technical replicates, not independent biological replicates. Thus, there was no possible way to estimate the plant-to-plant variability within a replication. In addition, the dynamics of pathogen populations, the changes of the phyllosphere microbiome and molecular defence responses such as the expression of defence-related genes, like phenylalanine ammonia-lyase (PAL) and pathogenesis-related (PR) proteins were not directly examined.

## 6 Future directions

Repeated disease assessments at multiple time points (0, 7, 14, 21, 28 days after treatment) is recommended for future investigations to better characterize the disease progression and allow for calculation of the area under the disease progress curve (AUDPC). Taxon-specific evaluation of aphids, thrips and mites should also be included, including the appropriate measures of incidence, abundance and severity of damage, and identification to the appropriate taxonomic level. To determine the causal organism associated with the leaf-blight symptoms, pathogen isolation from symptomatic tissues, morphological characterization and molecular identification of the pathogen with appropriate DNA markers and sequence-based analyses should be done. If possible, pathogenicity testing should also be included, to further establish causal relationship. Individual, pairwise, three-way and complete consortium treatments should be tested along with appropriate dose-matched controls in a systematic factorial or combination-based experiments. These designs would allow formal evaluation of interactions between the consortium components, and would separate additive, synergic, and antagonistic effects, as well as formulation composition from effect of the dose of the consortium components. Treatment consistency should be confirmed under various environmental conditions and production systems through multi-season and multi-location trials. Further research is also warranted to assess fruit yield and quality, residue status and cost–benefit relationships to ascertain the practicality of microbial consortium-based disease management. Further mechanistic studies should include pathogen population dynamics, phyllosphere microbial communities and responses to defence including the expression of genes related to plant defence including PAL and PR proteins. Biochemical sampling at the individual plant level could also be included in future experiments to quantify plant-to-plant variability, rather than using pooled replication samples. These investigations would contribute to the development of a more sound understanding of the effectiveness, mechanism, repeatability and applicability of microbial consortium control of strawberry leaf blight.

## 7 Conclusion

In the present study, foliar application of microbial formulations, particularly the complete consortium at a total application rate of 10 mL L^−1^, showed promising performance in reducing the severity of Phomopsis leaf-blight symptoms and improving vegetative growth and selected biochemical defence traits of strawberry cv. Camarosa under the tested shade-net conditions. The higher total application rate generally resulted in stronger responses within the range tested, and the complete consortium showed favourable performance for several growth-related parameters. However, the individual consortium components were applied at one-quarter of their respective single-agent concentrations, and pairwise and three-way combinations were not included. Therefore, the observed consortium performance cannot be specifically attributed to synergistic interactions.

The complete microbial consortium at a total product input of 10 mL L^−1^ represents a promising treatment option for protected strawberry production under the conditions tested. Its potential incorporation into integrated pest management (IPM) programmes should be considered preliminary until validated through multi-season and multi-location trials, including assessment of fruit yield, fruit quality, economic feasibility, and appropriately designed combination experiments with dose-matched controls.

## Data Availability

The raw data supporting the conclusions of this article will be made available by the authors upon request, without undue reservation.
